# Radiographic Changes After Pubic Symphysis Plating and Their Clinical Relevance: An Exploratory Longitudinal Cohort Study

**DOI:** 10.3390/life16050730

**Published:** 2026-04-28

**Authors:** Adrian Claudiu Carp, Bogdan Veliceasa, Awad Dmour, Ștefan Șelaru, Ștefan-Dragoș Tîrnovanu, Mihnea-Theodor Sîrbu, Bogdan Puha, Norin Forna, Liliana Savin, Alexandru Filip, Dragoș-Cristian Popescu, Paul-Dan Sîrbu

**Affiliations:** Grigore T. Popa University of Medicine and Pharmacy, 700115 Iasi, Romania; adrian-claudiu.carp@umfiasi.ro (A.C.C.); dmour-awad@umfiasi.ro (A.D.); sselaru@gmail.com (Ș.Ș.); stefan-dragos.tirnovanu@umfiasi.ro (Ș.-D.T.); mihnea-theodor.sirbu@umfiasi.ro (M.-T.S.); bogdan.puha@umfiasi.ro (B.P.); norin.forna@umfiasi.ro (N.F.); liliana.savin@umfiasi.ro (L.S.); alexandru-filip@umfiasi.ro (A.F.); dragos.popescu@umfiasi.ro (D.-C.P.); paul.sirbu@umfiasi.ro (P.-D.S.)

**Keywords:** pubic symphysis, pelvic ring injury, pubic symphysis plating, mechanical failure, radiographic loosening, postoperative widening

## Abstract

Background: Pubic symphysis plating is a common method for stabilizing traumatic pubic symphysis disruptions, yet reported rates of implant failure vary widely in the literature. This variability may reflect inconsistent definitions and failure to distinguish clinically significant early construct failure from later asymptomatic postoperative radiographic changes. Methods: We performed a retrospective observational study of 30 patients with traumatic pubic symphysis disruption without associated fractures of the pubic body or pubic rami treated with open reduction and plate fixation. Pubic symphysis distance (PSD) was measured on admission CT, immediate postoperative anteroposterior pelvic radiographs, and follow-up CT scans obtained at 3, 6, and ≥12 months. Early mechanical failure, qualitative radiographic signs of implant loosening, and radiographic loss of reduction were predefined. Non-parametric tests were used to compare patients with and without early mechanical failure and to evaluate longitudinal PSD changes; analyses of potential associated factors were exploratory. Results: Early mechanical failure occurred in 4 patients (13.3%) within 30 days and presented as an acute symptomatic event with imaging-confirmed construct compromise requiring revision. In exploratory univariable analysis, early failure was more frequent in female patients and in those with obesity or osteoporosis, although these findings should be interpreted cautiously given the very small number of events. PSD changed significantly over time (*p* < 0.001), with minimal increase during the first 3 months, greater widening between 3 and 6 months, and little additional change thereafter. Qualitative radiographic signs of implant loosening and widening were observed in 8 patients (26.7%) during follow-up without clinically documented pain, instability, or need for revision. No clear association was demonstrated between PSD widening and final functional outcome measured by the Majeed score, although these analyses were limited by sample size and wide confidence intervals. Conclusions: In this retrospective cohort, postoperative radiographic widening and qualitative signs of implant loosening were not by themselves associated with clinically evident failure requiring revision during the available follow-up. Early failure was identified by acute clinical symptoms with imaging-confirmed construct compromise, whereas delayed widening was often observed without clinically documented pain, instability, or reoperation. These findings suggest that postoperative imaging should be interpreted together with symptoms and overall pelvic stability, while recognizing the methodological limitations of the study.

## 1. Introduction

The pubic symphysis is a fibrocartilaginous joint reinforced by strong ligamentous structures that allow limited translational (2–3 mm) and rotational (1–2°) motion, thereby contributing to physiological pelvic flexibility during gait and, in females, permitting widening of the pelvic ring during childbirth [1,2,3]. Disruption of these stabilizing structures typically results from high-energy trauma and is often associated with severe concomitant injuries and increased mortality [2,3,4,5]. Surgical reduction and stabilization aim to restore pelvic ring alignment and stability, facilitate early mobilization, and improve functional recovery [6,7,8]. Among the available techniques, external fixation and symphyseal plating are commonly used methods for temporary or definitive stabilization of the anterior pelvic ring [9,10,11,12,13].

Despite the widespread use of symphyseal plating, reported rates of fixation or implant failure vary considerably in the literature, ranging from 12% to 75% [14,15,16]. Similarly, significant postoperative loss of symphyseal reduction has been described in 7% to 88% of cases, suggesting persistent difficulty in maintaining stable alignment after surgery. Early hardware failure has been reported in approximately 10–15% of anteroposterior compression pelvic injuries and may require revision surgery [14,15,16,17,18].

At the same time, many studies have described frequent postoperative imaging findings such as screw loosening, plate deformation, or progressive widening of the symphyseal gap in patients who remain asymptomatic. This raises the question of whether all postoperative radiographic changes represent clinically significant mechanical failure. The absence of consistent definitions, together with the frequent grouping of early symptomatic construct failure and later asymptomatic radiographic changes under the same label of “implant failure,” may partly explain the wide variability in reported failure rates. Importantly, in many reports, failure has been defined primarily by imaging criteria, without clear correlation to symptoms or revision requirement.

The primary objective of this study was to characterize the longitudinal evolution of pubic symphysis distance after plate fixation and to evaluate whether postoperative radiographic changes correspond to clinically relevant mechanical failure. We hypothesized that progressive postoperative widening may follow a consistent temporal course and may not necessarily reflect clinically significant failure. By integrating imaging findings with clinical follow-up, this study aims to provide a more clinically meaningful framework for interpreting postoperative changes after symphyseal plating.

## 2. Materials and Methods

### 2.1. Study Design and Patient Selection

This retrospective observational study included consecutive adult patients treated for traumatic pubic symphysis disruption between 1 October 2015, and 31 December 2022, at the Trauma Department of the University Hospital in Iași, Romania. All procedures were performed by the same senior surgeon.

**Figure 1:**
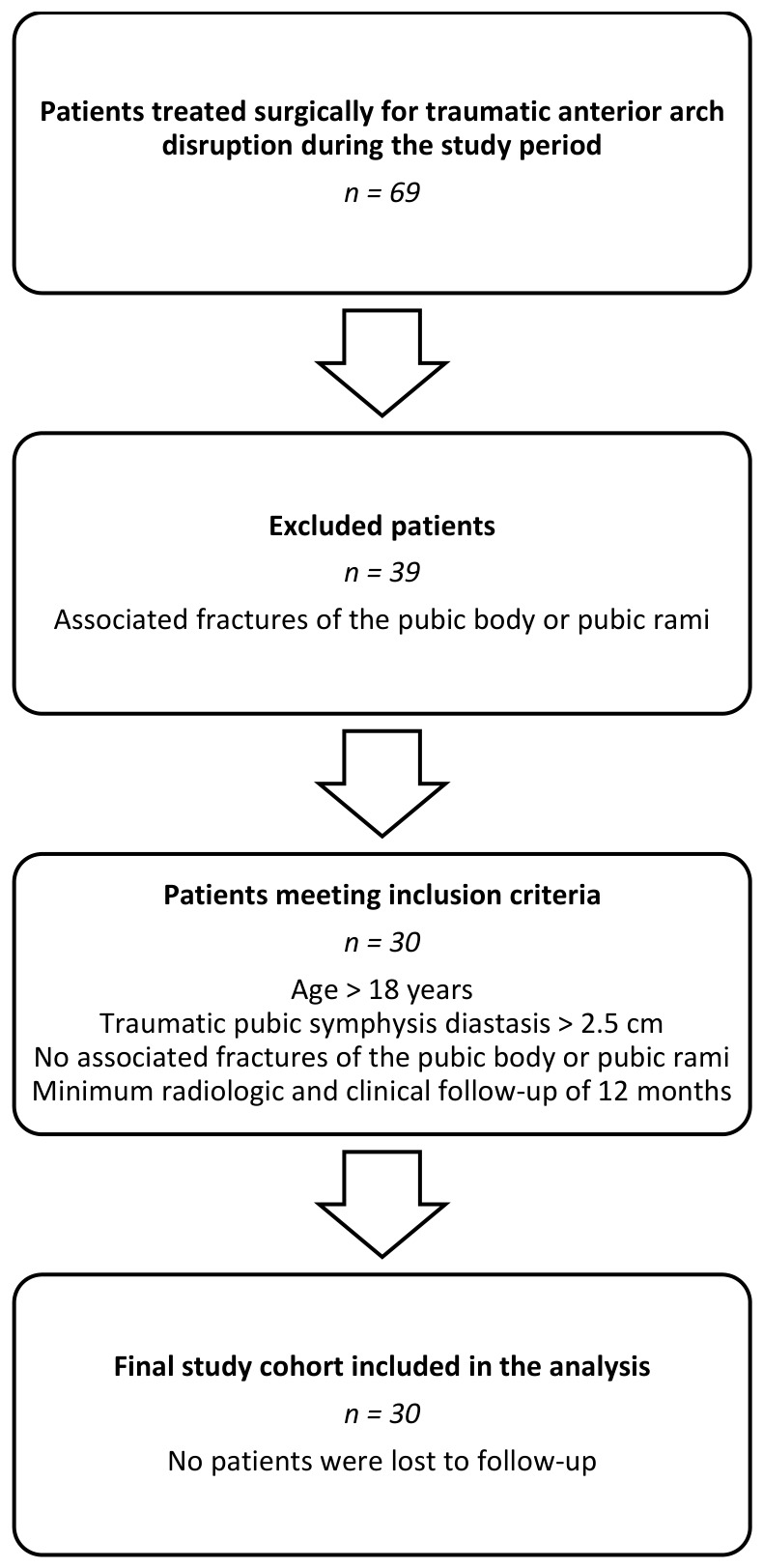
Flowchart of patient selection.

During the study period, 69 patients underwent open reduction and internal fixation (ORIF) of the pubic symphysis. Patients were eligible for inclusion if they were older than 18 years, had traumatic pubic symphysis diastasis greater than 2.5 cm, had no associated fractures of the pubic body or pubic rami, and had a minimum radiologic and clinical follow-up of 12 months. These strict criteria were intentionally applied to obtain a biomechanically more homogeneous cohort of patients with pubic symphysis disruption without associated anterior pubic arch fractures and to reduce the confounding effect of associated anterior pelvic ring fractures on symphyseal behavior after fixation. The findings should therefore be interpreted primarily in relation to this specific injury subset and may not be generalizable to more complex combined anterior pelvic ring injuries. Thirty patients fulfilled all inclusion criteria and constituted the final study cohort. The patient-selection process is summarized in Figure 1. This represented 43.5% of all patients treated surgically for symphyseal disruption during the study period.

All patients provided informed consent for clinical treatment. Ethical approval for the retrospective analysis of anonymized clinical and imaging data was granted by the institutional ethics committee. Because of the retrospective design and the use of anonymized data, the requirement for additional informed consent for study participation was waived by the ethics committee.

Fracture patterns were classified according to the Tile [19] and Young-Burgess [20] classifications.

### 2.2. Imaging Assessment and Measurement Protocol

All patients underwent standardized imaging at admission, including pelvic radiographs and computed tomography (CT) scans. Immediate postoperative assessment of reduction was performed using standardized anteroposterior (AP) pelvic radiographs. During follow-up, patients underwent serial imaging according to the institutional protocol for pelvic ring injuries, including pelvic radiographs and CT scans at approximately 3 months, 6 months, and ≥12 months. Follow-up CT examinations were also used for the present imaging analysis.

Pubic symphysis distance (PSD) was measured at five timepoints: at presentation, immediately postoperatively, and at 3 months, 6 months, and ≥12 months. Preoperative and follow-up PSD measurements were obtained on axial CT images using RadiAnt DICOM Viewer (version 2025.1, Medixant, Poznań, Poland). On CT, PSD was measured on the axial slice where both opposing pubic cortices and the symphyseal joint space were most clearly visualized simultaneously. The measurement was defined as the shortest cortical-to-cortical distance perpendicular to the joint line.

Immediate postoperative PSD was measured on standardized AP pelvic radiographs as the shortest distance between the opposing pubic cortices at the level of the symphysis. Because the immediate postoperative measurement was obtained using radiography rather than CT, this timepoint was used primarily as a practical clinical reference for reduction quality and for subsequent assessment of interval widening, rather than as a precise tomographic baseline.

All measurements were independently performed by two orthopedic specialists and one orthopedic resident according to a predefined measurement protocol. The observers were not involved in the surgical procedures. Because this was a retrospective study and PSD represented a relatively simple linear measurement performed according to a predefined protocol, formal interobserver agreement analysis using ICC or kappa statistics was not performed. This should be considered a methodological limitation.

### 2.3. Surgical Technique and Fixation Strategy

All patients were positioned supine on a radiolucent table. A Pfannenstiel approach was used to expose the pubic symphysis. After reduction under fluoroscopic guidance and direct visualization, fixation was achieved using either a 3.5 mm precontoured reconstruction plate with 4 or 6 holes or a dedicated pubic symphysis plate. Implant configuration, including plate type and length, was selected according to surgeon preference and intraoperative assessment. Because of the limited sample size, implant configuration was not analyzed separately as a predictor of outcome. Fluoroscopy was used intraoperatively to confirm satisfactory reduction and implant position.

In patients with Tile type C injuries or when posterior instability was identified intraoperatively, additional posterior pelvic ring fixation was performed using sacroiliac screws or plate-based posterior stabilization, depending on fracture pattern and surgeon preference.

### 2.4. Postoperative Management and Follow-Up Protocol

Antibiotic prophylaxis consisted of a single intraoperative dose of a third-generation cephalosporin. Thromboprophylaxis with low-molecular-weight heparin was administered for 35 days. Drains were removed after 24 to 48 h. Early mobilization and physiotherapy were initiated on postoperative day 2.

Weight bearing was restricted for 6 weeks, followed by gradual progression to full weight bearing at 10 to 12 weeks. Patients were followed clinically and radiographically at 6 weeks, 3 months, 6 months, and ≥12 months. Immediate postoperative reduction was assessed on standardized AP pelvic radiographs. Follow-up CT scans obtained at 3 months, 6 months, and ≥12 months as part of the institutional protocol for pelvic ring injuries were also used for symphyseal measurements in the present study. These CT examinations were part of routine clinical follow-up and were not performed specifically for research purposes.

Symptoms, perceived instability, and the need for reintervention were documented during follow-up; however, no standardized serial pain scale or functional outcome instrument was collected at each intermediate timepoint. Functional outcome was assessed only at final follow-up using the Majeed score [21].

### 2.5. Definitions of Postoperative Imaging Findings and Complications

Before statistical analysis, operational definitions were established to ensure consistent classification of postoperative findings.

For the purpose of this study, three postoperative phenomena were predefined:

Qualitative radiographic signs of implant loosening: screw halo, screw back-out, or plate deformation observed on follow-up imaging, without symptoms and without need for revision. Because this retrospective dataset did not include standardized quantitative thresholds for radiolucent zone width, screw angle change, or displacement, this variable should be interpreted as a qualitative imaging descriptor rather than a validated quantitative endpoint.

Radiographic loss of reduction: increase in PSD greater than 5 mm compared with the immediate postoperative measurement.

Acute symptomatic mechanical failure: acute construct failure characterized by sudden pain and/or mechanical symptoms, with imaging confirmation of plate or screw pull-out and need for revision surgery.

Early mechanical failure was defined as acute symptomatic mechanical failure occurring within the first 30 postoperative days.

The threshold of 5 mm for radiographic loss of reduction was selected as a pragmatic operational cutoff based on reported physiological symphyseal mobility and previously published radiographic criteria [22,23]. This threshold has also been used in prior clinical literature evaluating additional loss of reduction after traumatic pubic symphysis disruption [23]. However, no universally accepted threshold for clinically relevant postoperative loss of reduction currently exists, and this definition should therefore be interpreted as study-specific and operational rather than definitive.

Additional complications recorded included surgical site infection and the need for revision surgery.

### 2.6. Statistical Analysis

Statistical analysis was performed using SPSS software version 18.0 (IBM Corp., Armonk, NY, USA). Descriptive statistics and frequency analyses were calculated for all variables. Data distribution was assessed using the Shapiro–Wilk test and showed non-normal distribution for continuous variables; therefore, non-parametric tests were used throughout the analysis.

Comparisons between patients with and without early mechanical failure were performed using the Mann–Whitney U test for continuous variables and Fisher’s exact test for categorical variables. Given the very low number of early mechanical failure events, these univariable comparisons were performed only as exploratory analyses to identify possible signals within the cohort and were not intended to establish independent predictors. No multivariable modeling was feasible, and no adjustment for multiple comparisons was applied; therefore, these results should be interpreted with substantial caution.

Changes in PSD over time were analyzed using the Friedman test for repeated measures. Post hoc pairwise comparisons were performed using the Wilcoxon signed-rank test with Bonferroni correction. Given the relatively small sample size and the non-normal distribution of the data, a non-parametric repeated-measures approach was considered more appropriate than mixed-effects modeling for the longitudinal analysis performed in this study.

Correlations between imaging changes and functional outcomes were evaluated using Spearman’s rank correlation coefficient. Ninety-five percent confidence intervals for Spearman’s rho were estimated using bootstrap resampling (20,000 iterations). Interobserver reliability was not formally evaluated.

Because the immediate postoperative PSD was measured on AP pelvic radiographs whereas subsequent follow-up measurements were obtained on CT, longitudinal comparisons should be interpreted as imaging-based estimates of interval change and considered in light of potential modality-related measurement differences.

Because this was a retrospective cohort study, no a priori sample size calculation was performed. The study cohort was determined by the number of eligible patients treated during the study period who fulfilled the inclusion criteria.

## 3. Results

### 3.1. Study Cohort

Of the 69 consecutive patients treated surgically for traumatic pubic symphysis disruption, 39 were excluded because of associated fractures of the anterior pelvic arch. The remaining 30 patients fulfilled all inclusion criteria and constituted the study cohort.

The mean age was 51 years (range, 23–72 years). Male patients predominated (76.7%). Tile type C injuries were more frequent (73.3%) than type B injuries (26.7%). In accordance with the institutional treatment protocol, patients with Tile type C injuries, or those in whom posterior instability was identified intraoperatively, underwent additional posterior stabilization using either an ilioiliac screw, a posterior transiliac plate, or two V-shaped plates placed anterior to the sacroiliac joint. Road traffic accidents were the most common mechanism of injury (63.3%). Relevant comorbidities included obesity in 5 patients (16.7%), osteoporosis in 6 patients (20.0%), and diabetes mellitus in 9 patients (30.0%). The results are summarized in Table 1.

The mean initial pubic symphysis distance (PSD) at presentation was 40.81 ± 13.66 mm, and severe separation (>4 cm) was observed in 12 patients (40.0%).

According to the predefined criteria, radiographic loss of reduction greater than 5 mm relative to the immediate postoperative measurement was observed in 8 patients (26.7%) during follow-up. None of these patients had clinically documented pain, instability, or need for revision during routine follow-up. These cases were therefore interpreted as asymptomatic postoperative widening rather than clinically significant mechanical failure.

No patients were lost to follow-up.

### 3.2. Early Mechanical Failure: Exploratory Descriptive Comparisons

**Figure 2:**
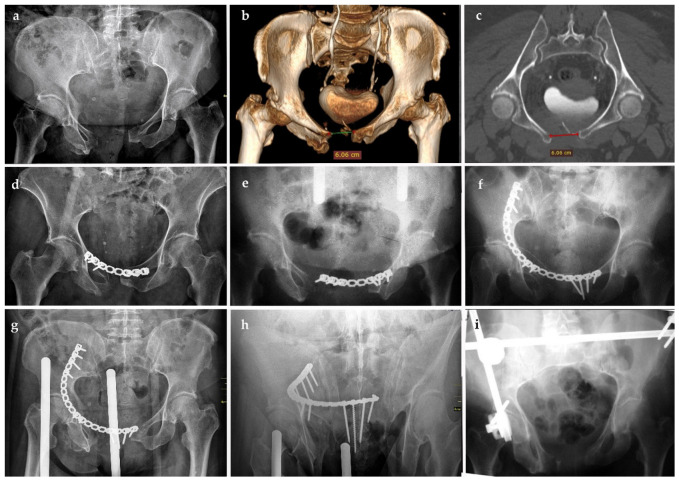
Example of acute symptomatic mechanical failure requiring revision surgery: preoperative pelvic radiograph (**a**), 3D CT reconstruction (**b**), and axial CT scan (**c**) showing severe pubic diastasis of 6.06 cm; immediate postoperative pelvic radiograph (**d**) showing adequate reduction in the pubic symphysis; radiograph obtained 4 days postoperatively (**e**) demonstrating pull-out of the plate and screws from the right pubic bone after sudden acute pain, consistent with early mechanical failure; post-revision radiograph (**f**) showing fixation with a longer reconstruction plate extending to the iliac wing; follow-up radiographs (**g**,**h**) showing contralateral plate and screw pull-out after a second acute painful episode; final radiograph after second revision (**i**) showing implant removal and stabilization with an external fixator.

Early mechanical failure, defined as clinically relevant construct failure requiring revision surgery within the first 30 days, occurred in four patients (13.3%). Exploratory univariable analysis showed that early mechanical failure occurred more often in female patients (*p* = 0.030), patients with obesity (*p* < 0.001), and those with osteoporosis (*p* = 0.018). No significant association was found with age (*p* = 0.40), initial PSD (*p* = 0.52), fracture type (*p* = 1.00), or diabetes mellitus (*p* = 0.56). Because only four early mechanical failure events occurred, these findings should be interpreted strictly as exploratory, statistically unstable, and hypothesis-generating observations rather than as reliable predictors of failure. Clinically, early failure presented with sudden pubic pain during mobilization, often accompanied by an audible “pop,” followed by imaging confirmation of plate pull-out. All four patients required revision surgery. One patient underwent two revision procedures because of recurrent construct failure (Figure 2).

### 3.3. Longitudinal Evolution of Pubic Symphysis Distance

Analysis of PSD measurements over time demonstrated a significant change across follow-up intervals (Friedman test, *p* < 0.001). The temporal evolution of PSD is summarized in Table 2.

From the immediate postoperative timepoint to 3 months, the mean increase in PSD was 2.83 ± 0.60 mm. A substantially greater increase was observed between 3 and 6 months, with a mean progression of 5.81 ± 1.66 mm. Beyond 6 months, additional widening was minimal (0.33 ± 0.06 mm), indicating relative stabilization thereafter.

Post hoc analysis showed significant interval differences, with the greatest change observed between 3 and 6 months (Wilcoxon signed-rank test with Bonferroni correction, *p* < 0.001).

### 3.4. Asymptomatic Postoperative Widening Versus Early Mechanical Failure

Qualitative radiographic signs of implant loosening and progressive widening of the symphyseal gap were observed in 8 patients (26.7%), predominantly during the 3- to 6-month interval. None of these patients had clinically documented pain, instability, or need for revision during routine follow-up.

**Figure 3:**
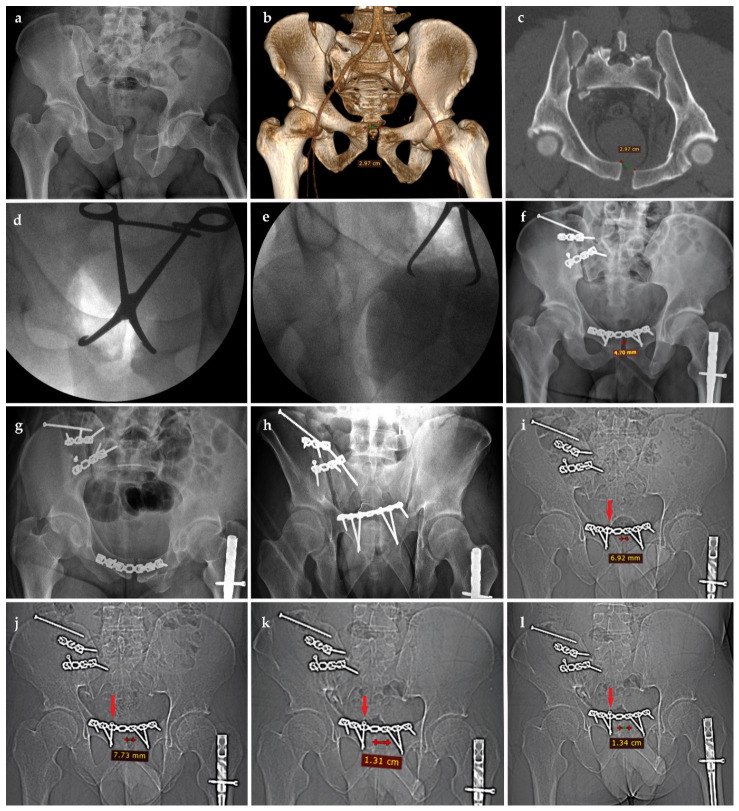
Example of progressive radiographic loosening and symphyseal widening without clinical symptoms: preoperative pelvic radiograph (**a**), 3D CT reconstruction (**b**), and axial CT scan (**c**) showing pubic diastasis of 2.97 cm; intraoperative fluoroscopic views (**d**,**e**) demonstrating reduction in the pubic symphysis; immediate postoperative pelvic radiographs (**f**–**h**) showing reduction in the pubic symphysis to 4.7 mm and restoration of pelvic alignment; follow-up CT at 45 days (**i**) showing loosening of the first screw on the left side of the pubic plate with widening of the symphysis to 6.92 mm; 3-month CT (**j**) showing further loosening and widening to 7.73 mm; 6-month CT (**k**) showing widening to 1.31 cm; 1-year CT (**l**) showing no substantial further progression. Despite these imaging findings, the patient remained asymptomatic and did not require revision surgery.

No specific radiographic threshold or imaging pattern reliably distinguished early mechanical failure from delayed widening. Instead, early failure was defined by acute clinical presentation with imaging confirmation, whereas delayed widening was observed in patients without clinically documented pain, instability, or need for revision during routine follow-up (Figure 3).

### 3.5. Functional Outcome and Correlation with Imaging Changes

At final follow-up, the mean Majeed score was 77.50 ± 14.29, corresponding overall to a good functional outcome.

Patients with Tile type B injuries had higher average Majeed scores than those with Tile type C injuries, although this difference did not reach statistical significance (*p* = 0.068, Mann–Whitney U test). No significant sex-related differences were observed. Although female sex was associated with early mechanical failure in exploratory univariable analysis, no significant sex-related difference was observed in final Majeed score across the overall cohort.

No significant correlation was identified between total PSD widening from the immediate postoperative measurement to final follow-up and final Majeed score (Spearman’s rho = −0.15, 95% CI −0.53 to 0.26; *p* = 0.42). Similar findings were observed when widening during the 3- to 6-month interval, which represented the period of greatest radiographic change, was analyzed separately (Spearman’s rho = −0.18, 95% CI −0.55 to 0.23; *p* = 0.34). However, these results should not be interpreted as proof of absence of association. Functional outcomes were assessed only at final follow-up, and the relatively small sample size may have limited the ability to detect moderate relationships between radiographic widening and long-term function.

### 3.6. Postoperative Complications

Postoperative complications included 3 surgical site infections (10.0%) and 5 revision procedures. Four patients experienced early mechanical failure requiring revision surgery. One of these patients subsequently required a second revision procedure because of deep surgical site infection associated with deterioration of the fixation construct; this ultimately required implant removal and stabilization with an external fixator.

All infections occurred in the early postoperative period and were managed with surgical debridement and targeted antibiotic therapy. No neurovascular injuries or thromboembolic events were recorded. Implant removal was not performed routinely and was reserved for symptomatic cases.

## 4. Limitations

This study has several important limitations. First, its retrospective single-center design introduces the possibility of selection bias and limits external validity. Second, the final cohort was relatively small, and only four cases of early mechanical failure were observed. This limited statistical power, precluded multivariable modeling, increased the risk of unstable exploratory associations, and reduced sensitivity to detect more moderate correlations between imaging findings and clinical outcomes. Third, the strict inclusion criteria were intentionally used to obtain a biomechanically more homogeneous anterior injury pattern by excluding patients with associated pubic body or pubic ramus fractures; however, this approach further reduced sample size and limits generalizability to more complex combined anterior pelvic ring injuries. Fourth, immediate postoperative PSD was measured on standardized AP pelvic radiographs, whereas preoperative and follow-up measurements were obtained on CT. Because immediate postoperative CT was not routinely available, formal radiograph-to-CT calibration or cross-validation could not be performed. Therefore, longitudinal comparisons should be interpreted as imaging-based estimates of interval change rather than as modality-independent quantitative measurements. Fifth, qualitative radiographic signs of implant loosening were recorded using predefined imaging descriptors rather than validated quantitative thresholds, and formal interobserver agreement analysis or blinded adjudication was not performed. Sixth, symptoms were documented during routine follow-up visits, but no standardized serial pain or functional outcome instrument was collected at the intermediate timepoints; functional outcome was assessed only at final follow-up. Seventh, dynamic radiographic assessments such as flamingo views were not routinely obtained, which limited detection of occult instability in patients with postoperative widening. Eighth, compliance with postoperative weight-bearing restrictions was monitored clinically but not objectively quantified, so the potential contribution of progressive loading to the observed widening pattern could not be formally assessed. Finally, although exclusion of associated pubic ramus and pubic body fractures improved biomechanical homogeneity of the anterior ring injury pattern, variation in posterior instability and posterior fixation quality could still have influenced anterior construct behavior and could not be analyzed separately. In addition, confidence intervals around the correlation estimates were wide, reflecting limited precision and reduced ability to exclude moderate effects. These limitations should be carefully considered when interpreting the findings.

## 5. Discussion

The main finding of this study is that postoperative radiographic changes after pubic symphysis plating were not, by themselves, associated with clinically evident failure requiring revision during the available follow-up. Early mechanical failure was consistently associated with an acute symptomatic presentation and imaging-confirmed construct compromise, whereas delayed radiographic widening and implant loosening were often observed in patients without clinically documented pain, instability, or reoperation. These findings suggest that imaging findings alone may be insufficient to define clinically meaningful failure and should be interpreted within the broader clinical context.

Importantly, the present study did not identify specific radiographic features that independently distinguished early failure from delayed widening. Instead, the differentiation between these entities was primarily clinical. This finding has direct implications for postoperative assessment, suggesting that reliance on imaging alone may lead to overestimation of failure rates and potentially unnecessary revision procedures.

Another important point of the present study is that imaging alone did not define clinically significant mechanical failure. Rather, the diagnosis of early mechanical failure depended on the combination of acute clinical presentation and imaging-confirmed loss of construct integrity. The value of the longitudinal imaging analysis was different: it allowed characterization of the postoperative evolution of pubic symphysis distance over time and demonstrated that delayed widening may follow a consistent temporal trend without clinically documented pain, instability, or reoperation during the available follow-up. This distinction may help explain the wide variability in reported rates of “implant failure” after pubic symphysis plating.

Previous studies have reported early hardware failure rates of approximately 10% to 15% after pubic symphysis plating [14,15,16], but many reports do not clearly distinguish acute symptomatic construct failure from later radiographic changes. In the present cohort, early mechanical failure occurred in 4 patients (13.3%), which is consistent with prior literature. In exploratory univariable analysis, early failure was more frequent in female patients and in those with obesity or osteoporosis. However, these findings should be interpreted cautiously. Because only four early failure events occurred, statistical power was limited and the risk of type I error must be acknowledged. These associations should therefore be considered exploratory and hypothesis-generating rather than definitive evidence of causal predictors.

The longitudinal imaging analysis showed a significant change in pubic symphysis distance over time, with the greatest widening occurring between 3 and 6 months, followed by little additional change thereafter. This temporal pattern should be interpreted cautiously. Because postoperative weight bearing increased during this period and compliance was not objectively quantified, the observed widening may reflect progressive loading, construct relaxation, healing-related remodeling, or a combination of these factors. The present study cannot determine the underlying mechanism. Accordingly, this pattern is best interpreted as a descriptive temporal radiographic finding rather than proof of a specific biomechanical adaptation process.

Radiographic implant loosening and widening of the symphyseal gap have been reported in up to 75% of patients after plating [15,24], often without clinical symptoms [15,25]. Our findings are consistent with these observations, but add a longitudinal perspective. In the present study, asymptomatic widening most commonly became evident between 3 and 6 months and showed minimal progression thereafter. Importantly, these radiographic changes were not associated with pain, instability, or need for revision surgery. This supports the interpretation that, in at least some patients, delayed widening may represent a postoperative radiographic course without clinically evident failure requiring revision rather than unequivocal clinically significant mechanical failure.

This distinction may help interpret some of the heterogeneity in the literature, but it is unlikely to be the only explanation. Several studies have reported frequent radiographic loosening without clear clinical consequence [15,18,25], whereas others have shown poorer outcomes when radiographic loss of reduction was accompanied by symptoms and clinically significant mechanical failure [23]. Differences across studies may also reflect variation in cohort composition, posterior ring injury severity, posterior fixation strategy, implant configuration, bone quality, reduction accuracy, and rehabilitation protocols. Therefore, our findings should be interpreted as complementary to prior reports rather than as a definitive contradiction of studies that found worse outcomes in patients with loss of reduction.

No significant correlation was identified between total PSD widening from the immediate postoperative measurement to final follow-up and final Majeed score (Spearman’s rho = −0.15, 95% CI −0.53 to 0.26; *p* = 0.42). Similar findings were observed when widening during the 3- to 6-month interval, which represented the period of greatest radiographic change, was analyzed separately (Spearman’s rho = −0.18, 95% CI −0.55 to 0.23; *p* = 0.34). However, these results should not be interpreted as proof of absence of association. The confidence intervals around the correlation estimates were wide, indicating limited precision and insufficient power to exclude a moderate relationship between postoperative widening and long-term function. In addition, functional outcome was assessed only at final follow-up, which limited evaluation of the temporal relationship between imaging changes and clinical recovery.

The reported variability in fixation failure rates after pubic symphysis plating is likely multifactorial. Differences in the definition of failure are probably a major contributor, but other factors may also influence outcome, including the quality of posterior pelvic ring stabilization, reduction accuracy, implant configuration, bone quality, body habitus, and adherence to postoperative rehabilitation protocols. These variables affect load transfer across the pelvic ring and the mechanical environment of the anterior fixation construct. In the present cohort, posterior stabilization was performed in all Tile type C injuries according to institutional protocol, but subtle differences in posterior reduction or fixation quality may still have influenced anterior construct behavior. Because of the limited sample size, implant configuration was not analyzed as an independent predictor of outcome, and larger studies would be required to evaluate these factors more reliably.

In addition, the absence of immediate postoperative CT imaging limits the ability to exclude subtle residual malreduction, which may have influenced subsequent radiographic widening.

Despite these limitations, the study has several strengths. It examined a biomechanically homogeneous cohort of patients with isolated anterior symphyseal disruption without associated pubic ramus or pubic body fractures, used predefined operational definitions, and combined longitudinal imaging with clinical follow-up. This design allowed for more precise characterization of postoperative symphyseal behavior than is available in many prior series.

### 5.1. Clinical Implications

The findings of this study have several practical implications. First, early symptomatic mechanical failure should be recognized as a clinically significant event characterized by sudden pain and imaging-confirmed construct compromise, and it requires prompt evaluation for revision. Second, progressive widening and qualitative radiographic signs of implant loosening observed during follow-up should not automatically be interpreted as definitive failure in the absence of symptoms. In our current clinical practice, we employ a watchful waiting approach for asymptomatic widening, including cases with widening of up to 13 mm in the present cohort, while prioritizing the patient’s clinical status-particularly the absence of pain, subjective instability, or need for reintervention-over purely radiographic findings. However, this does not mean that widening should be ignored. Isolated widening on routine imaging should prompt continued clinical surveillance and individualized reassessment of pelvic stability, with consideration of additional diagnostic testing when widening is progressive, symptoms develop, posterior instability is suspected, or overall stability remains uncertain. Third, the exploratory associations observed with female sex, obesity, and osteoporosis should be regarded only as preliminary signals requiring confirmation in larger cohorts. Overall, the present findings support interpreting postoperative imaging within its clinical context rather than using imaging alone to determine the need for reoperation.

### 5.2. Future Perspectives

Future research should aim to validate these findings in larger prospective multicenter cohorts with standardized postoperative imaging protocols and serial clinical outcome assessment. Further studies should also evaluate whether implant configuration, posterior pelvic ring stabilization, bone quality, body habitus, and adherence to rehabilitation protocols influence the likelihood of early mechanical failure or delayed asymptomatic widening. In addition, dynamic radiographic assessment and more detailed longitudinal functional evaluation may help clarify whether specific patterns of postoperative widening reflect benign postoperative adaptation or clinically relevant residual instability.

## 6. Conclusions

In this retrospective cohort, postoperative radiographic widening and qualitative signs of implant loosening after pubic symphysis plating were not by themselves associated with clinically evident failure requiring revision during the available follow-up. Early failure was identified by acute clinical symptoms with imaging-confirmed construct compromise, whereas delayed widening was often observed without clinically documented pain, instability, or reoperation. These findings support interpreting postoperative imaging within its clinical context, while recognizing that the study design, sample size, mixed imaging modalities, and absence of dynamic stability testing limit definitive conclusions. Accordingly, the present results should be considered hypothesis-generating rather than definitive clinical guidance.

## Figures and Tables

**Table 1:** Demographic, clinical, and injury characteristics of the study cohort (*n* = 30).

Variable	Value
Age (years), mean ± SD (range)	51.0 ± 14.6 (23–72)
Sex, *n* (%)	Male 23 (76.7%), Female 7 (23.3%)
Tile classification, *n* (%)	Type B 8 (26.7%), Type C 22 (73.3%)
Mechanism of injury, *n* (%)	RTA 19 (63.3%), Fall 5 (16.7%), Crush 5 (16.7%), Other 1 (3.3%)
Initial PSD (mm), mean ± SD	40.81 ± 13.66
Severe separation > 4 cm, *n* (%)	12 (40.0%)
Obesity, *n* (%)	5 (16.7%)
Osteoporosis, *n* (%)	6 (20.0%)
Diabetes mellitus, *n* (%)	9 (30.0%)
Early mechanical failure (≤30 days), *n* (%)	4 (13.3%)
Radiographic screw loosening, *n* (%)	8 (26.7%)
Surgical site infection (SSI), *n* (%)	3 (10.0%)
Patients requiring revision surgery, *n* (%)	4 (13.3%)
Revision procedures, *n* (%)	5 (16.6%)
Majeed score, mean ± SD	77.50 ± 14.29

Abbreviations: PSD—pubic symphysis distance; RTA—road traffic accident; SSI—surgical site infection.

**Table 2:** Longitudinal evolution of pubic symphysis distance (PSD) across imaging follow-up.

Timepoint	PSD (mm), Mean ± SD	PSD (mm), Median [IQR]
Preoperative	40.81 ± 13.66	37.00 [30.20–45.30]
Immediate postoperative	5.02 ± 0.44	5.00 [4.70–5.20]
3 months	7.85 ± 1.03	7.70 [7.05–8.28]
6 months	13.66 ± 2.69	13.15 [11.80–14.70]
≥12 months	13.99 ± 2.74	13.45 [12.10–15.00]

Immediate postoperative PSD was measured on standardized AP pelvic radiographs; preoperative and follow-up measurements were obtained on CT. Abbreviations: PSD, pubic symphysis distance; SD, standard deviation; IQR, interquartile range.

## Data Availability

The original contributions presented in this study are included in the article. Further inquiries can be directed to the corresponding author.

## Abbreviations

The following abbreviations are used in this manuscript:

PSD	Pubic symphysis distance
ORIF	Open reduction and internal fixation
CT	Computed tomography
cm	centimeter
mm	millimeter
RTA	Road traffic accident
SSI	Surgical site infection
